# A State‐of‐Art Review of the Metal Oxide‐Based Nanomaterials Effect on Photocatalytic Degradation of Malachite Green Dyes and a Bibliometric Analysis

**DOI:** 10.1002/gch2.202300001

**Published:** 2023-04-27

**Authors:** Ehtisham Umar, Muhammad Ikram, Junaid Haider, Walid Nabgan, Muhammad Imran, Ghazanfar Nazir

**Affiliations:** ^1^ Solar Cell Applications Research Lab Department of Physics Government College University Lahore Lahore 54000 Pakistan; ^2^ Tianjin Institute of Industrial Biotechnology Chinese Academy of Sciences Tianjin 300308 China; ^3^ Departament d'Enginyeria Química Universitat Rovira i Virgili Av Països Catalans 26 Tarragona 43007 Spain; ^4^ Department of Chemistry Government College University Faisalabad Pakpattan Road Sahiwal Punjab 57000 Pakistan; ^5^ Department of Nanotechnology and Advanced Materials Engineering Sejong University Seoul 05006 Republic of Korea

**Keywords:** malachite green dye, morphology, nanosized metal oxides, photocatalysis, preparation

## Abstract

A wide range of hard contaminants in wastewater is generated from different industries as byproducts of the organic compound. In this review, various metal oxide‐based nanomaterials are employed for the photocatalytic removal of malachite green (MG) dye from wastewater. Some cost‐effective and appropriate testing conditions are used for degrading these hard dyes to get higher removal efficiency. The effects of specific parameters are considered such as how the catalyst is made, how much dye is in the solution at first, how much nanocatalyst is needed to break down the dye, the initial pH of the dye solution, the type of light source used, the year of publications, and how long the dye has to be exposed to light to be removed. This study suggests that Scopus‐based core collected data employ bibliometric methods to provide an objective analysis of global MG dye from 2011 to 2022 (12 years). The Scopus database collects all the information (articles, authors, keywords, and publications). For bibliometric analysis, 658 publications are retrieved corresponding to MG dye photodegradation, and the number of publications increases annually. A bibliometric study reveals a state‐of‐art review of metal oxide‐based nanomaterials' effects on photocatalytic degradation of MG dyes (12 years).

## Graphical abstract

This review article describes the photocatalytic technique, reaction mechanisms, kinetics, recyclability, and stability in literature from 2017 to 2022 to degrade malachite green (MG) dye using various metal‐oxide‐based nanomaterials. The bibliometric analysis looks at the publication, citations, author collaboration to evaluate research impact, identify trends, countries, and which institutes are working on the MG dye.

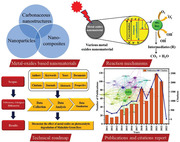

## 1 Introduction

Water is one of the basic sources for a human to survive on earth. Among the total availability of water resources, only 1% of water is utilized for human consumption.^[^ ^1^ ^]^ The contaminants from industries, like dye manufacturing, apparel, paper pulp mill, reed mat, tanneries, and printing significantly threaten our natural resources.^[^ ^2^ ^]^ The textile industries release a considerable amount of waste, that is, the organic dyes, chemicals, heavy metals, and oil in water bodies create a mass disaster for the environment because of their nasty nature and act as an agent for cancer disease.^[^ ^3^ ^]^ Textile dyes are highly soluble in water; due to this high release of dark dye, the entry of sunlight is blocked, causing severe damage to aquatic organisms and humans in and around the areas of these industries.^[^ ^4^ ^]^ These dyes are ≈10 000 in number. Viewing large‐scale usage, the azo dye comprises immense and critical damage.^[^ ^5^ ^]^

The malachite green (MG) dye “*N*‐methylated diaminotriphenylmethane” is an organic compound containing a “green” color crystal having mostly metallic luster used in various dyestuff industries and is one of the most effective chemicals worldwide.^[^ ^6^ ^]^ It is highly toxic; therefore, it must be purified before being released into the environment. It poisons the liver and causes damage to kidneys, gills, intestines, and damage to the mammalian cell. It may lead to cancer when inhaled; causes skin irritation with pain when it comes into contact.^[^ ^7^, ^8^ ^]^ The direct discharge of MG dye in the hydrosphere causes an imbalance in the environment. This untreated water used for irrigation purposes might result in reducing the quality of crop production.^[^ ^9^ ^]^ These dyes are banned in many countries but are still in use due to their low cost, accessibility, and potency.^[^ ^10^ ^]^

Treating these dye wastes before discharging them into water bodies is the most crucial need to be performed. One in eight people on the earth lacks domestic and clean drinking water. Around 1 million population has no access to pure water for drinking purposes. In developing countries, 3.5 million people die annually from inadequate sanitation and hygiene. Around 1.5 million children die because of water‐borne diseases. In 2025, around 1.8 billion populations will live in countries or areas where there is not enough water to go around. This water scarcity problem will make a worldwide systematized hazard.^[^ ^11^ ^]^ Researching inappropriate materials and perfect treatment methods/devices should be urgently implemented to overcome this issue.

This review contains the process in literature from 2011 to now (2022) for the degradation of MG dye by a photocatalytic technique using a variety of metal oxide nanoparticles (NPs).

A bibliometric study is a quantitative study that uses manuscripts as a database.^[^ ^12^ ^]^ This work used bibliometric methods to examine the current state of MG dye research from 2011 to 2022 including research fields, key researchers, levels, significant institutions, and countries‐wise research work. To sum up, the search results on the subject from the most reliable search engine, that is, Scopus, were summarized, and bibliometric studies were employed from the data.

In this current research, title search was done as part of a bibliometric analysis between 2011 and 2022 using VOSviewer with R‐studio and CiteSpace software to analyze a Scopus CSV and RIS file for countries, keywords, inter‐country coauthor networks, journals, and including co‐occurrence visualizations. Professor Chaomei Chen created the CiteSpace software at the start of 2004. The “Co‐occurrence” network maps of “keywords,” “authors,” “nations,” “institutions,” and subject categories, including co‐citation networks of “cited references,” “cited authors,” and “cited journals,” characterize it.^[^ ^12^, ^13^ ^]^

## 2 Malachite Green Dye Remediation Methods

**Figure 1:**
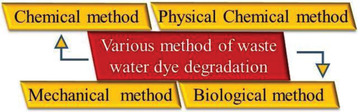
Various methods of treating industrial waste.

Both potable and wastewater should be treated for the benefit of society and to address the issue of insufficient drinking water supply. Diverse physical, chemical, and biological methods have been used to eliminate water pollutants (**Figure** **1**). The electrocoagulation process,^[^ ^14^ ^]^ adsorption, enzyme degradation,^[^ ^15^ ^]^ membrane filtration, ion exchange, chemical precipitation, chemical oxidation, flocculation,^[^ ^16^ ^]^ bacterial decomposition, electrochemical decolorization, ozonization^[^ ^17^ ^]^ nano photocatalysis, ceramic nanofiltration membrane, biofilms,^[^ ^18^ ^]^ organic resin, electrolysis, reverse osmosis, hybrid materials, oxidation, electrolysis, and biofouling.^[^ ^19^ ^]^ The qualities of these hazardous dyes reduce the efficacy of conventional decolonization techniques. However, the light‐based adsorbent (photocatalysis) approach has proven highly effective at degrading these dyes.

## 3 Remediated by Photocatalysis of Malachite Green Dye

The photocatalytic treatment uses heterogeneous photocatalysis, a semiconducting absorbent that breaks down many pollutants when exposed to light including organic pollutants in the atmosphere. The photocatalysts can completely degrade dye in a short amount of time, even at room temperature. More importantly, no toxic residues are left behind, and the organic pollutants are completely broken down into water and carbon dioxide, which are not dangerous byproducts.^[^ ^20^ ^]^ The efficiency of degradation is also affected by the conditions of the photocatalysis reaction, such as the light source used for irradiation (sunlight is a natural light source), the time in which light irradiation is, the pH value of the dye solution, the amount of catalyst needed for efficient degradation, the distance between the light source and the dye, the temperature during the reaction, and the concentration of the dye solution.^[^ ^21^ ^]^ At first, activated carbon, carbon nanotubes (CNTs), mesoporous silica, and chitosan beads, which are all porous and smaller than a nanometer, were used as adsorbents. Some drawbacks of these materials were that they were hard to use, did not work as well, were expensive, and needed a lot of energy. As a photocatalyst strongly suggested, different materials were compensated for the abovementioned problems.^[^ ^22^ ^]^

## 4 Nanomaterials

In material science and chemistry, nanotechnology is an essential technique. Consequently, the results of advanced nanotechnology led to the invention of nanomaterials (NMs) with exclusive and unexplored features.^[^ ^23^ ^]^ Several NMs, including TiO_2_ metal oxide, Ag nanoparticles, carbon nanotubes, and discreet aqueous fullerene (nC_60_), have demonstrated potential antibacterial activity and water treatment. This study revealed that antimicrobial NMs are biocompatible.^[^ ^24^ ^]^

**Figure 2:**
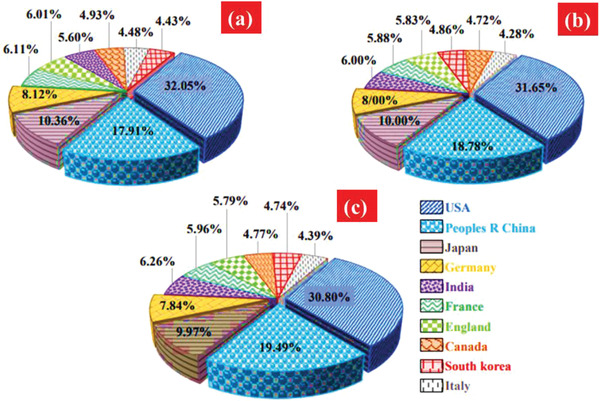
Top ten manuscript writing nations in nanomaterials and their compared contribution % to one another during 1975–2020: a) “carbonaceous nanostructure,” b) “nanoparticle,” and c) “nanocomposite.” Some keywords were chosen “carbonaceous nanostructures,” “nanoparticles,” “nanocomposites,” “wastewater,” and “treatment” (databases of WOS). Reproduced with permission.^[^ ^25^ ^]^ Copyright 2019, Royal Society of Chemistry.

In **Figure** **2**, the top ten nanotechnology‐based publications and their relative contribution percentages from 1975 to 2020: a) “carbonaceous nanostructure,” b) “nanoparticle,” and c) “nanocomposite.” Carbonaceous nanostructure, nanocomposite, wastewater, nanoparticle, and treatment were selected as keywords in Web of Science (WOS) databases.^[^ ^25^ ^]^

**Figure 3:**
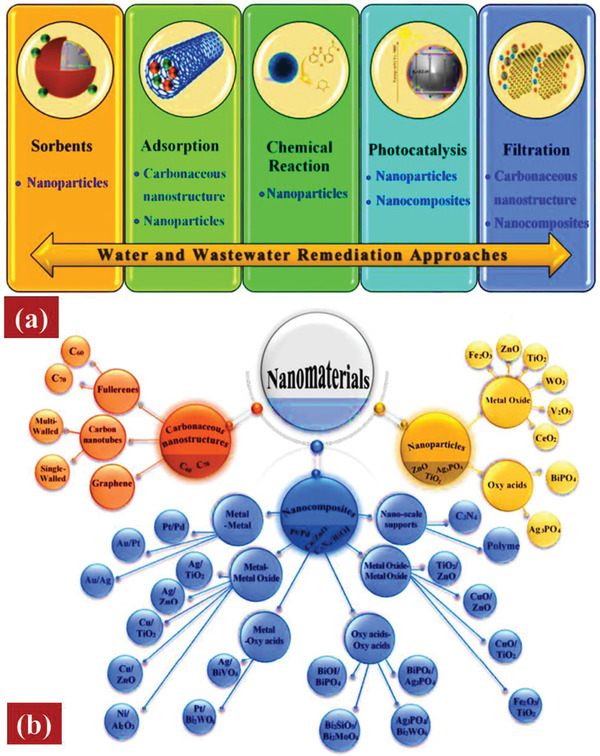
a) Common ways to remediation water, groundwater, and wastewater. b) Sorting out of the various NMs utilized to treat water and 150 wastewater goals. Reproduced with permission.^[^ ^25^ ^]^ Copyright 2019, Royal Society of Chemistry.

Various nanomaterials include carbon nanostructures, oxide nanoparticles, metal nanoparticles, and semiconducting nanoparticles (**Figure** **3**a). Nanoparticles have the potential to cause damage to microbial cells through a variety of mechanisms, including oxidizing cell components, inhibiting trans‐membrane electron transmission, interfering with or entering the cell envelope, and producing secondary products as reactive oxygen species (ROS) or aqueous heavy metal ions.^[^ ^26^ ^]^ The photocatalysis mechanism involves light energy to accelerate chemical reactions, producing reactive species that can degrade pollutants or synthesize useful products.^[^ ^27^ ^]^ In the past, “nano‐metals,” such as Zn, Ag, Mg, and Cu, were utilized to treat diseases; by manipulating the physicochemical features of these NMs, non‐toxic and effective antibacterial medicines for human health can be manufactured.^[^ ^28^ ^]^ The remediation of aquatic environments using five primary pathways is also significant, as shown in Figure 3a.

### 4.1 Carbonaceous Nanostructures

Carbon is one of the essential elements that may utilize to synthesize various carbon products; it is also one of the most abundant elements in the universe. The adsorption capacity of typical adsorbents like activated carbon is limited; to overcome this problem, scientists are trying to synthesize carbonaceous nanostructures. Additionally, carbon contributes to the development of carbon‐based nanostructures, including fullerenes (0D), “carbon nanotubes” (1D), and “graphene” (2D). Carbonaceous nanostructures are a deterministic base in water treatment because of their functional properties. These characteristics include high mechanical strength, a remarkable aspect ratio, strong thermal and electrical properties, impressive “hydrophobicity adsorption,” application simplicity, and separation characteristics.^[^ ^29^, ^30^ ^]^ Fossil fuels based on hydrocarbons, such as ethane and methane, are the primary source of these elements,^[^ ^31^, ^32^ ^]^ as described below in Figure 3b.

### 4.2 Nanoparticles

One of the essential categories of NMs is NPs, which are synthesized atomic and molecular clusters found in metals and the oxides of certain metals. Most of these atoms are quasi; as a result, they have a strong propensity to engage in chemical reactions with other atoms. According to the findings, 40% of the atoms are found within 10 nm above the surface; however, 20% are only found within 20 nm inside.^[^ ^33^ ^]^ This results in bioremediation, high adsorption capacity for metal ions, degradation, and reduction of pollutants from aqueous solution. Nanoparticles have become popular for various environmental contaminants cleaning purposes^[^ ^34^, ^35^ ^]^ such as nitrate,^[^ ^36^, ^37^ ^]^ pesticides,^[^ ^38^ ^]^ heavy metals, MG dyes, hydrocarbons,^[^ ^39^, ^40^ ^]^ radioactive elements, and salts.^[^ ^41^ ^]^ The two major categories of nanoparticles are metal oxides and oxy acids, as shown in Figure 3b.

### 4.3 Nanocomposites

Solid structure nanocomposites are classified into two fundamental categories: I) an inorganic solid core surrounded by organic shells or vice versa, and II) a mixture of two or more inorganic/organic phases.^[^ ^42^, ^43^ ^]^ Combinations of both inorganic and organic phases are characterized as the third form of solid structure nanocomposites. Impregnating metal nanoparticles into porous substrates (such as polymers and C_3_N_4_) was utilized to synthesize composite adsorbents.^[^ ^44^ ^]^ In recent years, combining nanoparticles into a single nanocomposite facility beneficial changes in their functionality (synergistic association effects) has gained much importance.^[^ ^45^, ^46^ ^]^ Generally, nanocomposites are obtained by combining metals, metal oxides, and oxy acids in the following combinations: metal–metal, metal oxides–metal oxides, metal–metal oxide, metal oxides‐oxy, acids‐oxy acids, and acids. Different classifications of nanocomposites are represented in Figure 3b.

## 5 Metal Oxide Nanoparticle as Photocatalyst

Different catalysts have been synthesized to improve photocatalytic activity. Metal oxide nanoparticles are less toxic and easily modified into hydroxides or oxides. Another important property is its band gap between observing the oxidation and reduction process. The band gap of nanomaterial affects their photocatalytic activity, with a smaller band gap corresponding with higher photocatalytic activity. An increase in absorption accompanied redshift, manifesting that photocatalyst is time reliable and cost‐effective.^[^ ^47^ ^]^ Photocatalysts are often synthesized from nanoparticles of metal oxide such as TiO_2_, ZnO, Bi_2_O_3_, Fe_2_O_4_,^[^ ^48^ ^]^ WO_3_, CuO, Cu_2_O,^[^ ^49^ ^]^ SnO_2_, BiVO_4_, InTaO_4_, CeO_2_, Bi_2_WO_6_, ZnAl_2_O_4_, ZnGaNO, and Zn_1.7_GeN_1.8_O.^[^ ^50^ ^]^ These metal oxides have many shapes, such as nanoparticles, nanospheres, nanofibers, nanotubes, nanoribbons, and sheets.^[^ ^51^ ^]^ **Table** **1** shows how different metal oxide nanoparticles degrade MG dye and the conditions of the experiment.

**Table 1:** Malachite green dye degradation by various metal oxide‐based nanoparticles, experimental conditions, and degradation efficiency

Metal oxide NMs	Synthesis technique	Morphology	Photocatalytic experimental setup	Degradation efficiency [%]	Year	Ref.
ZnO	Wet chemical precipitation method	Nanoparticles like structure	0.02 g/100 mL, 100 Watt tungsten lamp, pH = 10, bandgap 3.37 eV	MG 94.14% (41 min); 80.15%, (61 min); 67.65% (61 min)	2016	[60]
Nano‐composite hydrogel	Polymerization/co‐precipitation	Irregular and rough‐nature	300 min, photocatalyst, and placed under UV lamp (6 W, 254 nm with pH = 7,	91	2017	[61]
ZnO:Ag	Soft chemical method	Spherical structure	45 min, 500 Watt tungsten lamp, 50 mg/50 mL,	88.8	2017	[62]
A_2_PdF_6_ (A = K, Rb)	–	Hexagonal structure	60 min, visible light, pH = 4.66, temperature of 30 °C, band gap of 2.67 eV	95	2017	[63]
ZnO	Sol–gel	Spherical structure	Sunlight, 45 min, 50 mg/50 mL	97	2017	[64]
SnIn_4_S_8_	Solvothermal method	Spherical structure	500 kW Xe lamp, 30 min, at room temperature, band gap 1.53 eV	Strong degradation	2017	[65]
RGo‐CoS	–	Small grain	Sunlight, 30 min, under sunlight with an average intensities of ≈450 W m^−2^, pH = 7, dye solution 50 mL, band gap ≈1.15 eV,	Maximum	2017	[66]
ZnO:Mg/RGO	Hummer's method	Hexagonal structure	Visible‐light, 75 min, 500 W tungsten visible‐lamp, 50 mg dye solution, band gap 3.22 eV	99.56	2017	[67]
BiOCl	Hydrolysis method	Tetragonal structure	Visible light, 120 min, pH 2.3 to 14, under normal room temperature, bandgap 3.2 eV	Remarkable	2017	[68]
TiO_2_: Fe	Sol–gel method	Nanotubes, orthorhombic	Sunlight, 210 min, band gap 2.57 eV	Complete	2017	[69]
CeO_2_	Chemical precipitation method	Cubic fluorite	Visible light, 80 min, bandgap value 2.90 eV	99	2017	[70]
TiO_2_	Micro‐emulsion method	Spherical	10 mg catalyst, 10 ppm dye, visible light, 50 min, band gap 3.2 eV	96.40	2018	[71]
Mn‐doped ZnO	Wet diffusional impregnation	Tetragonal	3 g catalyst, 80 ppm concentration, visible light, 180 min, band gap 3.2 eV	Faster degradation	2018	[72]
ZnO/CuO	Hydrothermal method	–	0.2 g catalyst, 15 ppm dye, pH‐10, UV lamp, 240 min, band gap value 4.42 eV	82	2018	[73]
ZnO	Wet chemical method	Flower‐like structure	60 min, pH 5, high temperature (25 to 35 °C), band gap value 3.37 eV	88	2018	[74]
Zinc‐oxide	Co‐precipitation method	Flower‐like shape	10 ppm, 180 min, band gap value 3.31 eV	95	2018	[75]
Cu_2_O	Chemical deposition method	Cubic shape	50 ppm dye, 15 mg photocatalyst, and placed under UV lamp (6 W, 254 nm with pH = 8, band gap 2 eV	99	2018	[76]
Ti‐BALDH	Chemical precipitation method	Spherical structure	5 mg photocatalyst, 1 × 10^−4^ m dye, 250 W tungsten halogen lamp, 120 min, pH = 3, band gap	95	2018	[77]
Zn, Mg)xCu1‐xBi_2_O_4_	Hydrothermal method	Flower‐like structure	60 min, pH = 3, solar light, band gap value 1.33 eV	Maximum degradation	2019	[78]
La_2_CuO_4_‐decorated ZnO	Green synthesis method	Non‐uniform spherical shape	2.5 mg photocatalyst, 25 mL dye, visible lamp (125 W) for 120 min, 1.137 eV	91	2019	[79]
Xanthan gum /SiO_2_	Ultra‐sonication with polymerization	Lobule	10 mg catalyst, 450 ppm of MG dye, pH = 7, the temperature of 30 °C, 480 min,	99.5	2019	[80]
CuMn_2_O_4_	Co‐precipitation method	Flake‐like structure	Daylight, 60 min. UV light, 60 min, band gap value 2.54 eV	94.80, 90.10	2019	[81]
Chitosan/TiO_2_	–	Spherical nanoparticles	70 ppm, 90 min, band gap value ≈ 3 eV	90.70	2019	[82]
Iron oxide	Impregnation method	–	LED irradiation, 240 min 10–50 ppm of water, 1.5 g of catalyst, pH 4.6	97	2019	[83]
CSP‐GO‐TiO_2_	–	–	UV irradiation	69	2019	[84]
rGO/CuS	Co‐precipitation method	Irregular hexagonal	100 mg photocatalyst, 10 ppm dye, under sunlight at room temperature, band gap value 2 eV	97.60	2020	[85]
ZnFe_2_O_4_	Probe sonication	Spongy like	Under sunlight, UV lamp, it took about 180 min, 2.4 eV	98–88	2019	[86]
Mn_0.5_Cu_0.5_Cr_2_O_4_	Hydrothermal	Uniformly distributed and closely packed particles	10 ppm dye, under visible light with 40 mg of catalyst, H_2_O_2_, 40 min, band gap 1.37 eV	96	2020	[87]
Hematite	Combustion	Spherical and irregular structure	Presence of H_2_O_2_, 20 ppm dye, UV source of 250 W, 0.1 g catalyst, 70 min, band gap 1.45 eV	100	2020	[88]
Cu_2_O	Sonochemical method	Uniform Icosahedron	10 ppm dye, 10 mg catalyst, visible lamp, 45 min, 2.26 eV	91.89	2020	[89]
ZnO	Sol–gel method	Spherical structure	10 ppm dye concentration, 20 mg catalyst, UV lamp, 40 min, band gap 3.3 eV	99	2020	[90]
ZnO	Green synthesis method	Irregular hexagon	100 ppm dye, 10 mm catalyst, 150 min, band gap value 3.37 eV	Complete	2020	[91]
MgFe_2_O_4_@ CoCr_2_O_4_	Green synthesis method	Uniform spherical	fluorescent lamp (*λ* > 400, 90 W) 10 ppm dye solution, 0.05 g catalyst, natural pH, 90 min, 1.76 eV	92	2020	[92]
CuWO_4_‐RGO	Hydrothermal method	Agglomerated with polycrystalline nature	2 ppm dye, 50 mg catalyst, 370 W mercury halide visible light, 60 min, 2.2 eV	93	2020	[93]
CuWO_4_‐GO	Ball‐milling method	Microstructure	0.05 g catalyst, 10 ppm dye, visible lamp, 80 min,	95	2020	[94]
MnFe_2_O_4_	Microwave‐assisted combustion method	Irregular shape agglomerates	30 mg catalyst, 50 ppm dye, 60 min under natural pH condition	Maximum complete	2020	[95]
Magnetic nickel oxide	Precipitation method	Nano‐size grains	Visible light, room temperature,120 min,	57	2020	[96]
Chitosan–zinc oxide	Chemical Precipitation method	Hexagonal	pH of 8, 2.3 mg L^−1^, and dosage of 0.6 g and 180 min	98.5	2020	[97]
Fe‐Cu binary oxides	Electron spun method	Hair like structure	3 mg catalyst, 100 ppm dye solution, pH = 1, UV lamp, 60 min	91.40	2021	[98]
ZnO	Electrochemical vapor deposition method	Flower‐like morphology	Sunlight, band gap 3.3 eV, 160 min	99.99	2020	[99]
ZnO	Co‐precipitation method	Flower‐like structure	10 ppm dye, 0.5 mg catalyst, under UV irradiation (1000 ultraviolet cross‐linker, energy × 100 µ j/CM2), 180 min,	89	2020	[100]
CuO‐modified silicon nanowires	Electroless deposition technique	Nanowire	Room temperature, visible light (*λ* = 400–700 nm) or UV light (*λ* = 350–400 nm) or presence of PMS, 100 min, 1.2 eV	98	2020	[101]
ZnO and ZnO‐b‐cyclodextrin	Sol–gel method	Nanoparticle	0.01 g/10 mL, pH 7, wavelength range of 400–700 nm,	84%	2021	[102]
Cobalt Oxide	Sol–gel method	Spherical structure	50 mg nanocatalysts, 100 mL of 1 × 10–5 m dye, Xe lamp of 80 mW cm^−2^, 100 min	91.20	2021	[103]
Ag‐CdSe@GO	hydrothermal method	Nano‐sheets and nanorods	120 mg mL^−1^, 5 ppm, 25 mint, band gap 1.74 eV	97	2021	[104]
ZnO	Co‐precipitation method	Hexagonal structure	10 ppm dye, UV lamp, 180 min, 3.41 eV	89	2021	[105]
TiO_2_, ZnO	Synthetic method	Spherical rod shape	9 W UV lamp, pH 3, temp‐25 deg, 30 ppm dye, 16 min, 3.2 eV	79.4 and 97.5	2021	[106]
rGO‐Fe_3_O_4_/TiO_2_	Hydrothermal method	Spherical structure	100 ppm dye, 15 mg catalyst, 55 min, visible lamp, 2.6 eV	99	2021	[107]
Copper ferrite‐graphene oxide	Hydrothermal method	Hexagonal structure	0.01 g GO/CuFe_2_O_4_ was spread into 10 mL dye solution, 240 min,	90.70	2021	[108]
PVDF‐GO/black‐TiO_2_	–	Multi‐structural	10 ppm dye, 0.5 g L^−1^ of catalyst, pH of 2, 500‐W Halogen lamp, 30 min, 3.2 eV	74	2021	[109]
CuO	Green synthesis method	–	Visible light, bang gap 1.68 eV,	Complete	2021	[110]
NiO/TiO_2_, CuO/TiO_2_	Wet diffusional impregnation process	Tetragonal	Microwave irradiation (300–500 W), Various band gap, 160 min, 2 eV	Various degradations	2021	[111]
CuO	Precipitate method	Nanorod	10 ppm dye and 10 mg of catalyst,	92.40	2021	[112]
CQDs/CuO	Wet chemical method	Leaf‐like nanosheets	Under UV lights, 30 min, 1.2 eV	Maximum	2021	[113]
ZrO	Biosynthesis method	–	50 ppm dye, 0.01% catalyst, room temp, visible light, 45 min, 4.46 eV	Complete degradation	2021	[114]
Al/RGO/Ag	Layer‐by‐layer assembly technique	A large, wrinkled, and thin layered structure	20 mL of 1 × 10^−5^ dye, 1.02% catalyst, 3 min	80.9	2021	[115]
MAl_2_O_4_/activated carbon‐based composite	Chemical precipitation method	Irregularly dispersed surface	120 min, under visible light,	100	2021	[116]
NiZY	Impregnation method	Uniform cubic shape	200 mg photocatalyst, 10 ppm dye, under visible LED light (12 W), 460 nm, 60 min, Eg > 3 eV	96	2022	[117]
MoS_2_@TiO_2_@Au and MoS_2_@TiO_2_	Hydrothermal method	Nano‐sheets, and Nano‐flower	Under solar light irradiation (400–700 nm, 120 min, 3.2 eV	90.7	2022	[118]
SnO_2_ nanorods	–	Nanorod	15 min, visible light,	24.70	2022	[119]
NM‐BiFeO_3_	–	Composite	1000 mg L^−1^ solution of MG dye, light intensity (0, 25, 55, and 105 W), pH 3–9,	96	2022	[120]
Fe_3_O_4_/N‐Bi_2_MoO_6_	Simple hydrothermal method	Orthorhombic, nanoplate‐like structure	20 ppm photocatalyst, pH (4, 6, and 8), 60 min, absorption red shift	98.2	2022	[121]
Ag@AgCl and Ag@AgCl‐GO	Green synthetic method	Sphere‐like, microspheres	Sunlight, 350 s, room temperature, 3.89 eV	88 and 99	2022	[122]
MoS_2_/Mg(OH)_2_/BiVO_4_	Hydrothermal method	Nanorods shape, nanoparticles (nano‐composite)	Sunlight (25, 75, and 160 min), 2.27 eV	97, 68 and 90	2022	[123]
GO/formamide‐based GOLLC	Hummer method	Thin film	Higher concentration (2–300 mg mL^−1^), 120 min, source visible light, ≈ 2–2.5 eV	99.18	2022	[124]
CuO‐Gd_2_Ti_2_O_7_	Two‐phase fabrication method	–	Under a visible lamp in 90 min, 1.78 eV	86.60	2022	[125]
Co‐doped TiO_2_	Hydrothermal method	Agglomerated and non‐uniform	Sunlight, UV lamp, visible light, 5 mg Co_2_^+^‐TiO_2_, 180 min, 2.95 eV	82, 31, and 74	2022	[126]
WO_3_ decorated 2D graphene sheet	Microwave irradiation method	Highly porous 2D rGO sheets	UV lamp, 3.3 eV, under UV light shows maximum degradation, 0.1 g/100 mL	97	2022	[127]
Fe(III)‐Cross‐linked alginate‐carboxymethyl cellulose	–	Bead shape	pH = 4, 10 ppm dye, 0.1 g catalyst, UV lamp, 30 min, 2.0 eV	98.80	2022	[128]
CuO	Co‐precipitation method	Spherical	0.03 g catalyst, 50 mL dye, room temperature, 200 W Argon lamp,	94.26	2022	[129]
Gd and Fe doped LaNiO_3_	Micro‐emulsion method	Spherical nanoparticles	200 W Argon lamp, room temperature, 50 min, 1.96 eV	96.7	2022	[130]
TiO_2_–NiO	Sonochemical method	Spherical shape	20 wt% catalyst, UV light, 2.295 eV	High catalytic performance	2022	[131]
Aurivillius oxides nano‐sheets	Non‐hydrothermal method	Nanosheets	6.97 mg L^−1^, centrifuged at 4500 rpm for 15 min, under visible light, 3.27 eV	91	2022	[132]
ZnO	Co‐precipitation method	Hexagonal	Visible light, 120 min, a minimum dosage of 0.1 g catalyst, 3.42 eV	95	2022	[133]
GO	Hummers method	Nanoparticles	UV lamp, 90 min,	85	2022	[134]
Mn_3_O_4_	Ultra‐sound‐assisted method	–	101–162 mg catalyst, visible lamp	Maximum degradation	2022	[135]
Molecules Coated Silver Oxide Nanoparticle	(biosynthesis) Curcuma zanthorrhiza and HR‐LCMS	Nanoparticles spherical	10 mg L^−1^, band gap value 4.55 eV, under sunlight, 30 min	94.7	2022	[136]
TiO_2_‐inulin‐Fe_3_O_4_	Simple co‐precipitation method	–	Fluorescent lamp with 36 W power,	Better removal	2022	[137]
ZnO‐NPs	Green synthesis method	Hexagonal	320 min, under the ultraviolet light, band gap 3.3 eV	96	2022	[138]
TiO_2_‐GO	Sol–gel method	Nanosheet, homogeneous and aggregated like cluster	13 min, band gap (3.07 and 2.92 eV), pH = 13, 10 mg photocatalyst, 30 mL dye, visible light and room temperature	84	2022	[139]
Cr VI by TiO_2_/GO	Sol–gel method	Nanoparticles	pH 5–6, Eg = 2.92 eV, under sunlight, crystallite size 5.1 nm and 23 nm, 90 min	48	2022	[140]
*β*‐CD‐CuO/ZnO	Sol–gel method	Hexagonal shape particles	180 min, 0.05 g/50 mL photocatalyst, 1 × 10^−5^ m, pH = 7–10, visible light, 3.2 eV	71.43% and 79.90% using CuO/ZnO and *β*‐CD‐CuO/ZnO photocatalysts respectively	2022	[141]

## 6 Reaction Mechanism and Kinetics

**Figure 4:**
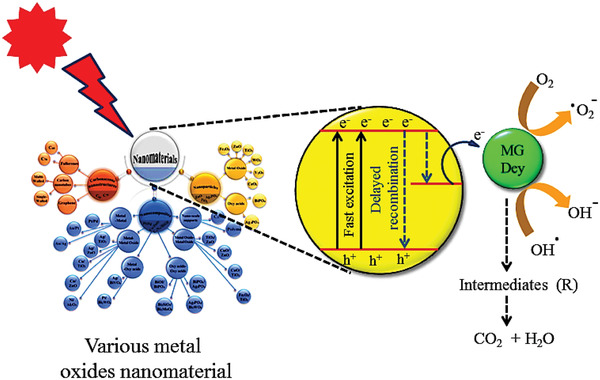
Mechanism for photocatalytic degradation of cationic malachite green dye over various metal oxides nanomaterial under various light irradiations. Reproduced with permission.^[^ ^25^ ^]^ Copyright 2019, Royal Society of Chemistry.

**Figure** **4** depicts a suggested mechanism for photocatalytic degradation of MG dye over metal oxide‐based nanomaterials under various light irradiation sources. The separation of electron–hole pairs and inhibition of the recombination rate is attributed to the improved photocatalytic behavior of NMs, which promotes and increases the number of electron–hole pairs involved in photodegradation. Various functional groups and the high surface‐to‐volume ratio of metal oxide‐based nanocomposites improve the interaction with dye molecules during photocatalytic dye degradations. The generated electrons (e^−^) interact with the dissolved oxygen molecule to form superoxide ion radicals (O_2_▪^−^). The hydroxyl radicals (▪OH+H^+^) are formed when positively charged holes (h^+^) combine with H_2_O. The dye degradation is usually caused by the active participation of ROS such as hydroxyl radical (OH•), superoxide radical anion (O_2_•^−^), and electrons–holes pairs.^[^ ^27^ ^]^

When the photon energy was larger than or equal to its band gap,^[^ ^52^, ^53^ ^]^ these electron–hole pairs shifted to the surface from the NMs. They interacted with the MG dye molecules to contribute to redox processes, and the dye molecules were transformed into harmless intermediates, H_2_O, and CO_2_. Under solar irradiation, this synergistic effect causes more electrons to promote from the valence band (VB) to the conduction band (CB).^[^ ^53^, ^54^ ^]^ Meanwhile, more electron vacancies are generated in VB, and thus the recombination process starts. As a result, with the help of the NMs photocatalyst, higher state (CB) electrons transfer to MG dye molecules and breakdown the MG dye (pollutant) to harmless intermediates H_2_O and CO_2_ molecules (Figure 4). The following equations describe the numerous photocatalytic degradation processes conceivable through the NMs catalyst

(1) $$\begin{array}{c} \mathrm{NMs}+\mathrm{hv}\;\to \mathrm{NMs}\left(e^{-}\right)+h^{+} \end{array}$$

(2) $$\begin{array}{c} H_{2}O+h^{+}\to \;{}^{•}\mathrm{OH}+H^{+} \end{array}$$

(3) $$\begin{array}{c} O_{2}+e^{-}\;\to O_{2}^{•-} \end{array}$$

(4) $$\begin{array}{c} O_{2}^{•-}+H^{+}\;↔{\mathrm{HOO}}^{•} \end{array}$$

(5) $$\begin{array}{c} 2\mathrm{HO}O^{•}\;↔H_{2}O_{2}+O_{2} \end{array}$$

(6) $$\begin{array}{c} H_{2}O_{2}\;\to 2OH^{•} \end{array}$$

(7) $$\begin{array}{c} \mathrm{Dye}\;+\;{\mathrm{OH}}^{•}\;\to {\mathrm{CO}}_{2}+H_{2}O\;\left(\mathrm{dye}\;\mathrm{intermediates}\right) \end{array}$$

(8) $$\begin{array}{c} \mathrm{Dye}+h^{+}\left(\mathrm{VB}\right)\;\to \mathrm{oxidation}\;\mathrm{products} \end{array}$$

(9) $$\begin{array}{c} \mathrm{Dye}+e^{-}\left(\mathrm{CB}\right)\to \mathrm{reduction}\;\mathrm{products} \end{array}$$

Porous structure distribution of material with a high surface area can offer substantial electro‐active sites and provides fast ion diffusion pathways; both are essential for photocatalytic applications.^[^ ^55^ ^]^ Additionally, the reactive sites on the surface of magnetic recyclable photocatalysts may considerably boost the effectiveness of decomposing any complicated dye molecules utilizing the photocatalyst.^[^ ^56^ ^]^

## 7 Recyclability and Stability Study

**Figure 5:**
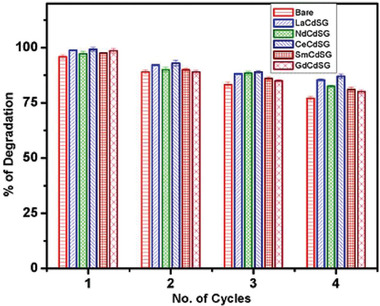
Catalytic stability and reusability of RE doped CdS nanoparticles on methylene blue degradation. Reproduced with permission.^[^ ^27^ ^]^ Copyright 2022, Elsevier.

Recyclability and stability were understood by an example of the dye used under the nanocomposite.^[^ ^27^ ^]^ Reusability without any loss in catalytic efficiency is one of the most crucial factors for wastewater treatment to reduce the catalyst cost.^[^ ^56^, ^57^ ^]^ Several cycles of photocatalytic degradation were used to test the generated CdS‐G nanocomposite's stability and reusability (**Figure** **5**). The nanocomposites were removed from the solution by centrifugation in each cycle, washed with methanol, and then dried at 70 °C in a vacuum oven. The dye degradation under sunlight was satisfactorily tested on all nanocomposites for up to four cycles. From Figure 5, it can be seen that the nanocomposite efficacy in preventing dye degradation remained steady up until the third cycle and that it has thereafter steadily decreased. After four consecutive cycles, the catalytic activity has maintained over 80% of its initial value, demonstrating the produced RE‐CdS‐G nanocatalyst's good stability and reusability up to four cycles within 90 min. Even after four cycles, Ce‐CdS‐G and La‐CdS‐G demonstrated considerable efficiency, with 87% and 85%, respectively. With an 80% degradation rate even after five cycles,^[^ ^57^, ^58^, ^59^ ^]^ reported the Ce‐TiO_2_ nanocomposites for degrading the dye under UV radiation.

## 8 Bibliometric Analysis

Bibliometric analysis studies publications in a particular field that provides current information about the subject. The investigation explains co‐citation, bibliographic coupling, research trends, co‐authorship networks, subjects, journals, and the growth of new fields. This review examines the most‐cited articles, the most‐used keywords, the evolution of publications, and the co‐authorship network. VOSviewer, R‐studio packages, and Citespce software were used. The statistical examination of published articles shows that connections between papers about a specific research topic or field by examining other published works have cited them on the same subject or in the same field.^[^ ^142^, ^143^ ^]^

**Figure 6:**
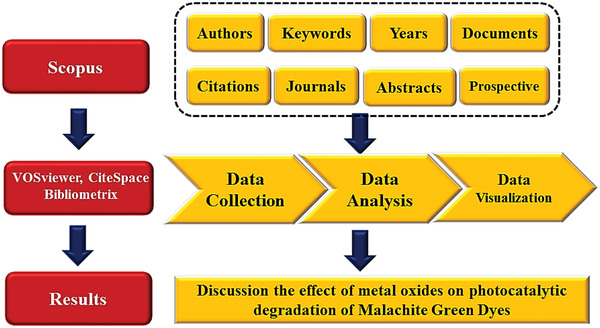
Technical roadmap for bibliometric analysis of the effect of metal oxides on photocatalytic degradation of MG dyes.

The pertinent information (including publication year, nation, author, journal, keywords, citations, and abstracts) for 658 articles has been downloaded. The CSV raw and RIS files were acquired from Scopus (as of 28 October 2022). A dataset consisting of the CSV raw and RIS files was employed to input the analysis findings into the Bibliometric software. The technical route map is depicted in **Figure** **6**.

**Figure 7:**
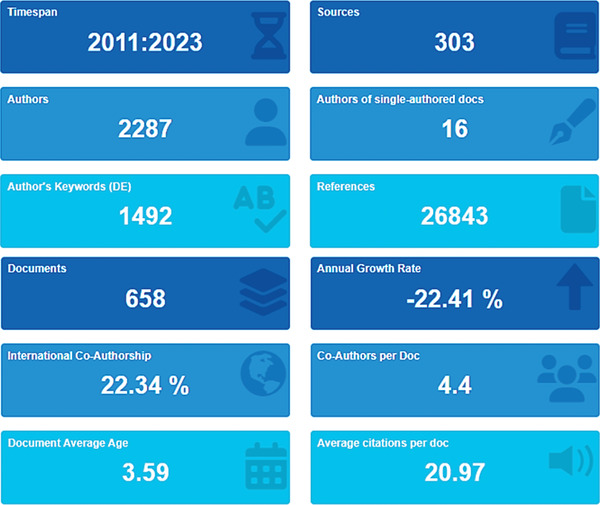
Basic information CSV raw file data were collected from Scopus (as of 28 October 2022).

Basic information about “Malachite green dye” data with their description and results were collected from Scopus (As of 28 October 2022). Within 12 years (2011–2022), data were filtered from 658 documents, 2 review articles, and 594 articles, with an average citation per doc of 20.97, international co‐authorship of 22.34%, references 26 843, author keywords (DE) of 658 proceedings as shown in **Figure** **7**. The main information data is collected from the Scopus search database from 2011–2022 (Figure 7).

### 8.1 Keywords Co‐Occurrence

**Figure 8:**
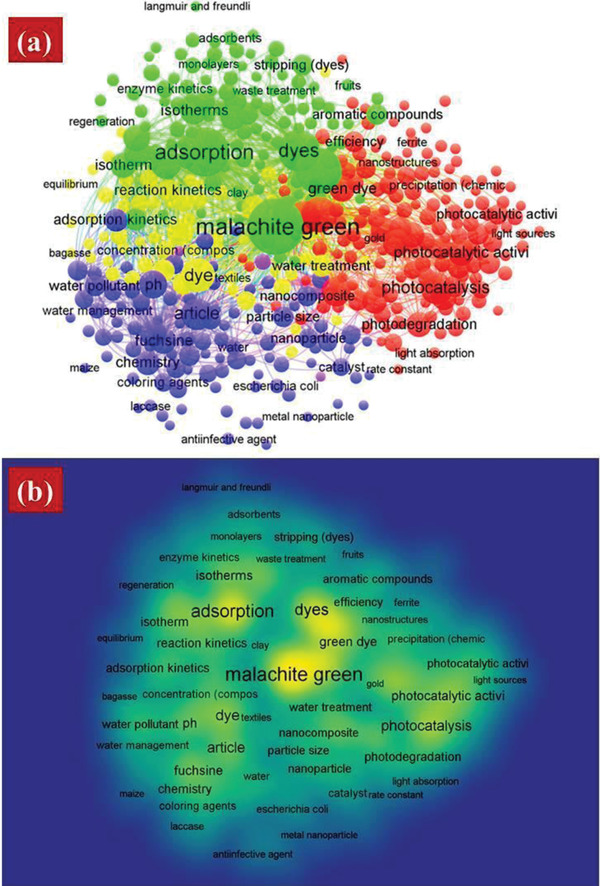
Cluster image of “keywords plus.” a) Network visualization, b) density visualization generated from CSV raw file data collected from Scopus (as of 28 October 2022).

The outcome of the cited journals, most‐cited articles, influential institutions, and cluster analysis was sorted and analyzed as part of the search strategy and method. There are several articles about how metal oxides affect the photocatalytic degradation of MG dyes. This is a significant trend in wastewater treatment, materials science, and metal oxide research. The most repeated keywords about MG dye degradation are shown in **Figure** **8**a,b, comprising about 5 total clusters; 435 items, 27 965 total links; 71 155 total link strengths. Cluster: 1 has 155 items, 407 total links, 2516 total link strength, and 128 occurrences; cluster 2 has 117 items, 434 total links, 5434 total link strength, and 402 occurrences; cluster 3 has 97 items, 361 total links, 2219 total link strength, and 90 occurrences, cluster 4 have 62 items, 357 total links, 2086 total link strength and 111 occurrences and cluster 5 have 4 items, 113 total links, 174 total link strength, and 5 occurrences.

The most‐important clusters (density of visualizations is centered) are the “red,” “green,” and “blue clusters.” The “red cluster” contains 155 total items; the most important items have occurrences (total links strength) as follows; scanning electron microscopy 128(407), Fourier transformation infrared 109(401), photocatalysis 92(274), X‐ray diffraction 75(357), nanoparticles 64(305), photocatalytic activity 58(229), green dye 55(276), synthesis (chemical) 53(277), degradation 52(276), photodegradation 51(253), zinc oxide 51(239), efficiency 32(213), transmission electron 29(246) and visible light 14(117). The green cluster have key words malachite green 401(434), carbonate minerals 242(218), adsorption 279(397), dye 183(410), kinetic 88(328), isotherm 67(276), thermodynamics 61(262), activated carbon 58(244), isotherm 50(269), adsorption capacities 49(250), initial dye concentration 38(234), solution 35(224), adsorbent 34(243) and compound 31(187).

The blue cluster have article 90(361), Ph 66(320), rosaniline dyes 58(295), fuchsine 56(295), chemistry 47(255), water pollutant 32(232), controlled study 32(256), nanocomposite 29(253), catalysis 28(218), nonhuman 27(219), unclassified drug 27(242), hydrogen‐ion concentration 25(209), coloring agents 25(195) and catalyst 23(199). The yellow cluster have dye 111(357), aqueous solution 60(307), wastewater treatment 53(297), reaction kinetic 39(262), water treatment 37(259), pollutant removal 37(229), malachite 35(231), response surface methodology 32(200), priority journal 24(238), optimization 32(185), concentration (composition) 22(180), biosorption 16(110) and textile 11(134). Finally the last purple color cluster have surface area 16(200), nitrogen 11(138), electron 5(113) and zeta potential 5(92). The importance of keywords for research is because they recognize and show the most important part of the research field.^[^ ^144^ ^]^ **Table** **2** shows the top 30 most prominent keywords in publications about this topic. These results will help future authors choose the Keywords that will facilitate research to find the information already published about a certain topic.

**Table 2:** Top 30 mostly used “keywords,” their “occurrences,” and “link strengths” in research results

S/N	Keywords	Cluster	Occurrences	Link strength
1	malachite green	2	401	434
2	carbonate minerals	2	242	218
3	adsorption	2	279	397
4	dye	2	183	410
5	scanning electron microscopy	1	128	407
6	cluster have dye	4	111	357
7	Fourier transformation infrared	1	109	401
8	photocatalysis	1	92	274
9	article	3	90	361
10	kinetic	2	88	328
11	X‐ray diffraction	1	75	357
12	isotherm	2	67	276
13	Ph	3	66	320
14	nanoparticles	1	64	305
15	thermodynamics	2	61	262
16	aqueous solution	4	60	307
17	rosaniline dyes	3	58	295
18	activated carbon	2	58	244
19	fuchsine	3	56	295
20	green dye	1	55	276
21	wastewater treatment	4	53	297
22	synthesis (chemical)	1	53	277
23	degradation	1	52	276
24	photodegradation	1	51	253
25	zinc oxide	1	51	239
26	isotherm	2	50	269
27	adsorption capacities	2	49	250
28	chemistry	3	47	255
29	reaction kinetic	4	39	262
30	initial dye concentration	2	38	234

### 8.2 Year‐Wise Publication and Citations Trend

**Figure 9:**
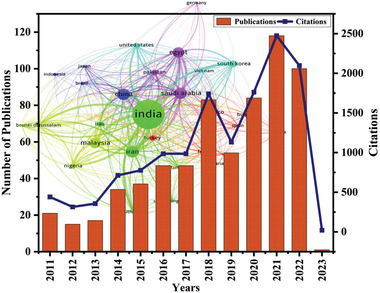
Citations and publications report on photocatalytic degradation of malachite Green dyes from 2011 to 2022, retrieved from Scopus with the keywords “Malachite green dye” and photocatalysis.

The number of publications and citations reported on “Malachite green dye” from the Scopus database (**Figure** **9**) indicates a developed interest in research areas, years‐wise publications, and citations. This demonstrated an increase in the number of research publications produced each year in this field. Consequently, the total number of citations reached more than 2474 in 2021, which has since decreased to more than 2097 in October 2022.

The results of publications and citations between 2011 and 2022 in the research area were not very famous until 2019. Consequently, the total publications and citations have increased several times than the previous. In recent years, photocatalytic activity has gained excellent economic and environmental viability and widespread acknowledgment from the scientific community. The significant development in investigating potential industrial wastewater replacements has gained worldwide attention.^[^ ^145^ ^]^ Within 12 years (2011–2022), data were filtered from 658 documents, 2 review articles, and 594 articles, average citation per doc of 20.97, international co‐authorship of 22.34%, references of 26 843, author keywords (DE) of 658 proceedings, as shown in Figure 9. The most productive authors in the field of photocatalytic degradation of MG dye with total citations and a total number of citations per year (**Table** **3**).

**Table 3:** The most successful 15 authors in the world photocatalytic degradation of MG dyes employing their author, title, total citations, and the total number of citations per year (as of October 2022)

Authors	Year	Title	Journal	Total citations	Total citation per year
AHMAD MA	2011	Removal of malachite green dye from aqueous solution using rambutan peel‐based activated carbon: equilibrium, kinetic and thermodynamic studies	Chemical Engineering Journal	301	25.083
SHARMA G	2015	Spion/*β*‐cyclodextrin core–shell nanostructures for oil spill remediation and organic pollutant removal from wastewater	Chemical Engineering Journal	117	14.1625
ABUKHADRA MR	2020	Photocatalytic degradation of malachite green dye using chitosan‐supported ZnO and Ce–ZnO nano‐flowers under visible light	Journal of Environmental Management	112	37.333
LIM LBL	2015	Application of *Casuarina equisetifolia* needle for the removal of methylene blue and malachite green dyes from aqueous solution	Alexandria Engineering Journal	109	13.625
SHARMA G	2016	Novel guar gum/Al_2_O_3_ nanocomposite as an effective photocatalyst for the degradation of malachite green dye	International Journal of Biological Macromolecules	109	15.571
LIM LBL	2015	Enhancing adsorption capacity of toxic malachite green dye through chemically modified breadnut peel: equilibrium, thermodynamics, kinetics and regeneration studies	Environmental Technology (UK)	100	12.5
SHARMA G	2017	Microwave‐assisted fabrication of La/Cu/Zr/Carbon dots trimetallic nanocomposites with their adsorptional vs photocatalytic efficiency for remediation of persistent organic pollutants	Journal of Photochemistry and Photobiology A: Chemistry	87	14.5
AHMAD MA	2012	Coconut (*Cocos nucifera*) shell‐based activated carbon for the removal of malachite green dye from aqueous solutions	Separation Science and Technology	69	6.273
BELLO OS	2012	Coconut (*Cocos nucifera*) shell‐based activated carbon for the removal of malachite green dye from aqueous solutions	Separation Science and Technology	69	6.273
LIM LBL	2014	Sorption characteristics of peat of Brunei Darussalam iv: equilibrium, thermodynamics, and kinetics of adsorption of methylene blue and malachite green dyes from aqueous solution	Environmental Earth Sciences	63	7
SHARMA G	2015	Nanocomposite pectin Zr (iv) selenotungstophosphate for adsorptional/photocatalytic remediation of methylene blue and malachite green dyes from aqueous system	Journal of Industrial and Engineering Chemistry	62	7.75
AHMAD MA	2012	Adsorptive features of banana (*Musa paradisiaca*) stalk‐based activated carbon for malachite green dye removal	Chemistry and Ecology	57	5.182
AHMAD MA	2014	Adsorptive removal of malachite green dye using durian seed‐based activated carbon	Water, Air, and Soil Pollution	53	5.889
LIM LBL	2019	Enhancing adsorption of malachite green dye using base‐modified *Artocarpus odoratissimus* leaves as adsorbents	Environmental Technology and Innovation	45	11.25
ABUKHADRA MR	2020	Insight into the photocatalytic properties of diatomite@Ni/NiO composite for effective photodegradation of malachite green dye and photo‐reduction of Cr (vi) under visible light	Journal of Environmental Management	44	14.667

### 8.3 Keyword Co‐Occurrence Timeline View

**Figure 10:**
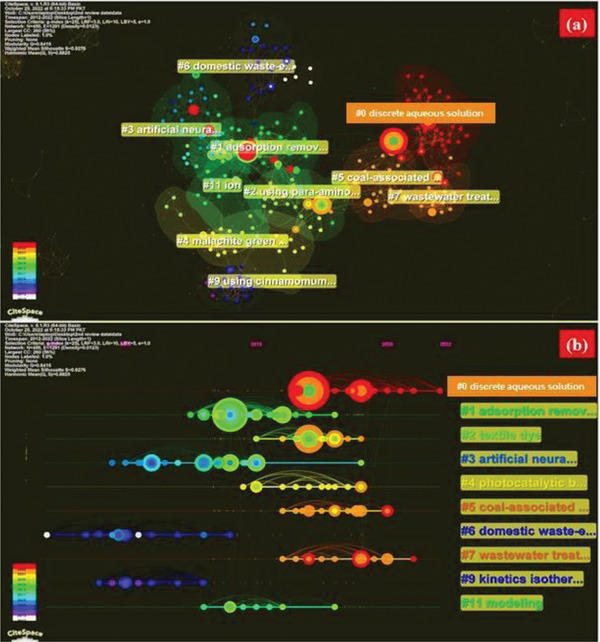
Maps of “keyword co‐occurrence” in publications from 2011 to 2022 on the effect of metal oxides on photocatalytic degradation of Malachite Green dyes. a) The “keyword co‐occurrence” cluster map. The names of the cluster indicate the key research areas. b) In the timeline view, you can see the “keyword co‐occurrence” cluster map. You can find the order in which the keywords in each cluster appear in time. The more nodes there are in clustering, the smaller the number.

**Figure** **10**a,b shows both the keyword co‐occurrence network and the keyword co‐occurrence cluster on maps. With a *Q*‐value of 0.8415, 12 clusters were found and a silhouette value was 0.9276. In **Table** **4**, the 12 largest groups are shown, and the timeline view shows that almost every year, there were new keywords (Figure 10b).

**Table 4:** The largest ten clusters of references in the co‐citation network

Cluster	Size	Silhouette mean	Year	Label (LLR)
0	44	0.886	2019	Discrete aqueous solution
1	39	0.932	2015	Discrete aqueous solution
2	31	0.914	2016	Using para‐aminobenzoic acid
3	29	0.909	2012	Artificial neural network modeling
4	28	0.951	2017	Malachite green synthetic dye
5	25	0.87	2017	Coal‐associated soil
6	23	0.986	2010	Domestic waste‐eggshell
7	15	0.985	2017	Wastewater treatment
9	14	0.996	2011	Using cinnamomum camphora sawdust
11	12	0.947	2015	Ion

We use a “cluster network” for all of the articles (from 2011 to 2022) in the field of “effect of metal oxides on photocatalytic degradation of MG dyes” (Figure 10). The co‐occurrence keyword knowledge of the graph shows which keywords are essential and used a lot. The size of each node is based on how references have been made recently. Co‐occurrence keywords can be divided into 12 sub‐clusters, including #0 aqueous solution, #1 adsorption removal, #2 using paper aminobenzoic acid, #3 artificial neural, #4 malachite green, #5 coal‐associated soil, #6 domestic wastewater, #7 wastewater treatment, #9 using Cinnamomum camphora sawdust, and #11 ion 42.

Figure 10b shows different Citespace timeline visualization.^[^ ^146^ ^]^ 12 clusters, with the number of citations in each and the names of the groups as shown in Figure 8b. When Citespace is observed, the co‐references only pick the 45 most‐used articles. All the references in Figure 10b were published between 2011 and 2022, which means there were not enough co‐references in terms of when they were published. The node's position shows the time it was published, the ring color shows the time it was cited, the ring thickness is based on how often it was cited, and the lines between the nodes show co‐references. With the spending of time, the color changes from green to orange and then changes to red.

A timeline view shows research trends have changed in various fields, and the timeline view observed that cluster nodes are connected with horizontal lines. The timeline view makes it clear how many nodes are in each cluster. More nodes mean that the field is more important. The most important articles in certain sub‐field show how to research topics that rise to popularity and are the most cited article. The timeline view shows the advancement of cluster 6 (domestic waste efflux) occurs first, indicating the previous studies of the photocatalytic effect of metal oxides. Cluster 0 (aqueous solution) and cluster 7 (wastewater treatment) developed later because they show how the early ideas improved (clusters 1, 2, and 3).^[^ ^147^ ^]^

### 8.4 Analysis of Coauthors' Country

**Figure 11:**
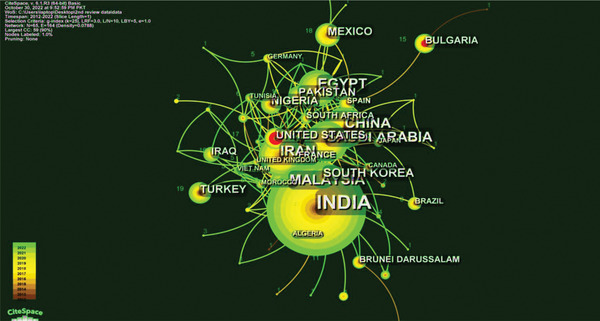
The joint map of the productive “countries.” Each node on the map stands for a different country. The different colors in the nodes showed when the articles were published. The more publications there are, the larger the area of the node. The more nodes there are in clustering, the smaller the number.

**Figure** **11** shows that the nodes are the countries, the lines are the productive countries that worked together, and 65 nodes and 164 linkages in international partners formed a network. The colors showed the year the articles were published, and the same rules apply to the colors of the lines. Figure 11 indicates that the publications of India, Saudi Arabia, Iran, China, Malaysia, Egypt, South Korea, and Turkey have progressed in the last 12 years. In this area, India, Saudi Arabia, Iran, and China often worked together with other countries.

**Table** **5** shows the 15 most productive countries in terms of publications. First, India has cited 247 publications, making it an essential part of this field. Saudi Arabia and Iran are second and third, with 62 and 58 publications, respectively. The quantity analysis of the publications shows two countries' research work led significantly. Iran, China, Malaysia, Egypt, and other countries at the end of the list have about the same number of articles.

**Table 5:** The top 15 most productive “countries”

S/N	Country	Number of publications
1	India	247
2	Saudi Arab	62
3	Iran	58
4	China	58
5	Malaysia	53
6	Egypt	52
7	South Korea	26
8	Turkey	19
9	Pakistan	19
10	Nigeria	18
11	Mexico	18
12	USA	17
13	Iraq	16
14	Bulgaria	15
15	France	10

## 9 Conclusion

This review article showed a widely used photocatalytic degradation approach to address the industrial wastewater issues, which posed a serious threat to society, and demonstrated how metal oxide nanoparticles might effectively handle MG dye degradation. Photocatalytic treatment can help society by resolving the lack of appropriate and safe drinking water. This bibliometric study examined publications' current status and trends between 2011 and 2022. To analyze the Scopus CSV and RIS files for countries, journals, inter‐country coauthor networks, and keywords, as well as co‐occurrence visualization, using VOSviewer, R‐studio, and CiteSpace software. The keywords extracted for the MG dye degradation contain a total of 5 clusters; 435 items, 27965 total links; 71155 total link strengths. Within 12 years (2011–2022), data were filtered from 658 documents, 2 review articles, and 594 articles citations per doc 20.97, international co‐authorship 22.34%, references 26 843, author keywords (DE) 658 proceedings. The top 15 most productive countries corresponding with articles were reported in this review. India is responsible for 247 publications and is a significant part of this field, and 62 and 58 records, Saudi Arabia and Iran are second and third, respectively.

## Conflict of Interest

The authors declare no conflict of interest.
